# Artificial intelligence in the detection of non-biological materials

**DOI:** 10.1007/s10140-024-02222-4

**Published:** 2024-03-26

**Authors:** Liesl Eibschutz, Max Yang Lu, Mashya T. Abbassi, Ali Gholamrezanezhad

**Affiliations:** Department of Radiology Division of Emergency Radiology, Keck School of Medicine, University of Southern California (USC), 1500 San Pablo Street, Los Angeles, CA 90033 USA

**Keywords:** Artificial intelligence, Deep learning, Retained Surgical bodies, Penetrating Injuries, foreign body ingestion, Tube malposition

## Abstract

Artificial Intelligence (AI) has emerged as a transformative force within medical imaging, making significant strides within emergency radiology. Presently, there is a strong reliance on radiologists to accurately diagnose and characterize foreign bodies in a timely fashion, a task that can be readily augmented with AI tools. This article will first explore the most common clinical scenarios involving foreign bodies, such as retained surgical instruments, open and penetrating injuries, catheter and tube malposition, and foreign body ingestion and aspiration. By initially exploring the existing imaging techniques employed for diagnosing these conditions, the potential role of AI in detecting non-biological materials can be better elucidated. Yet, the heterogeneous nature of foreign bodies and limited data availability complicates the development of computer-aided detection models. Despite these challenges, integrating AI can potentially decrease radiologist workload, enhance diagnostic accuracy, and improve patient outcomes.

## Introduction

Over the past decade, artificial intelligence (AI) has ushered in a new age of radiology and is poised to revolutionize medical imaging. The concept behind AI involves creating systems to perform tasks that typically require human intelligence. As the number and type of radiological imaging studies increase, so does the workload on radiologists globally. By automating routine tasks and providing rapid insights, AI can be a valuable tool in alleviating radiologist workloads.

Ultimately, AI holds great promise in the field of emergency radiology, particularly in the detection of foreign bodies. The ability of AI models to process vast amounts of imaging data quickly and accurately may enhance diagnostic accuracy in the imaging of non-biological materials. However, there is a paucity of literature describing the use of AI for this application, as well as a variety of other challenges. This review will delve into the various applications of AI in detecting non-biological materials, including retained surgical bodies, open and penetrating injuries, catheter and tube malposition, and foreign body ingestion and aspiration.

## Overview of artificial intelligence techniques

Within AI, machine learning (ML) techniques craft statistical models and algorithms to perform specific user-defined tasks [1]. This technique relies on expert knowledge to define and quantify radiographic features, which are then presented to the machine. Thus, machine learning trains itself to identify radiologic features based on patterns extrapolated from human-engineered data and algorithms [2]. Recent strides in AI have leaned heavily towards deep learning (DL), a subset of traditional machine learning techniques. Deep learning differs from traditional machine learning approaches as it uses a larger data set and doesn’t rely on human-engineered algorithms. Instead, it uses artificial neural networks (ANN) with hidden layers, such as convolutional neural networks (CNN), that permit a machine to train itself to perform a task [3]. Ultimately, DL systems can autonomously extract radiologic data from images, removing the human interface, manual image processing, and the risk of operator biases [4]. Thus, deep learning can outperform traditional machine learning when the data set is larger and more complex. The following sections will discuss the applications of deep learning techniques in detecting retained surgical bodies, open and penetrating injuries, catheter and tube malposition, and foreign body ingestion and aspiration.

## Retained surgical bodies

Retained surgical bodies (RSB), such as sponges, sutures, needles, and other instruments, can engender dire consequences for patients and cause severe financial and legal ramifications for the involved medical institution. These reportable “never events” are rare, with certain studies estimating an incidence of 1 in every 5,500–7,000 procedures, with higher rates with abdominal surgeries of up to 1 RSB per 1000 surgeries [5, 6]. The actual number of cases with RSB is most likely underestimated due to low reporting rates of these incidents, and patients can be asymptomatic and, thus, unaware of their occurrence. Many authors note that the risk of this complication decreases if institutions follow the recommended perioperative and postoperative checklists and guidelines [7]. Yet over 80% of operations noted to have RSB reported correct counts at the end of the case [8]. As most RSB have standardized shapes and sizes, computer-aided detection (CAD) systems can be highly effective for identification.

**Fig. 1:**
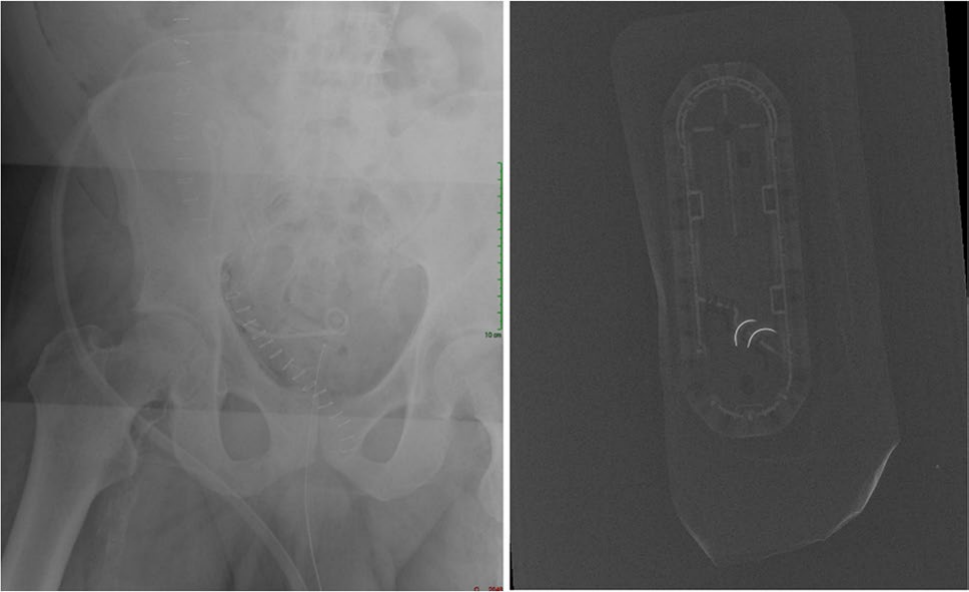
72-year-old male undergoing renal transplantation. Due to an incorrect count, an intraoperative X-ray was performed (A), which was negative for any retained metallic device. The optimal protocol in these clinical scenarios involves providing the interpreting radiologist an image of the missing foreign body (B).

Regarding the current imaging techniques to evaluate RSB, plain radiographs represent the gold standard imaging modality. On X-ray, retained objects often present as radiopacities with associated mass effect, mottled air, or density over surrounding soft tissues [9]. One benefit of this modality is that most sponges have radiopaque markers that make them detectable on X-ray [10]. However, these markers can become disfigured within the patient’s body, so they are not a reliable detection source [11]. Sponges without these markers are often visualized through cross-sectional imaging or radiographic visualization of radiolucency secondary to air trapping [10]. Yet it is essential to note that false-negative radiographs can exist, with certain authors reporting that intraoperative radiographs can miss up to one-third of RSB (Fig. 1) [12]. Further, obtaining and reading a radiograph can be time-consuming, particularly after a surgical case. Thus, AI techniques can play a prominent role in quickly and accurately detecting RSB.

Other imaging modalities such as ultrasound, CT, and magnetic resonance imaging (MRI) have also been proposed to identify RSB. On ultrasound, the most common presentation of retained surgical bodies such as sponges and gauze are hyperechogenic masses with hypoechoic rims [13]. Notably, ultrasound is minimally effective in identifying retained surgical bodies. In a study by Modrzejewski et al., the authors reported that ultrasound could detect one in 25 RSB cases, thus yielding a sensitivity of 4% [14]. Conversely, CT is the most sensitive detection method and is usually obtained if an X-ray returns negative and there is high clinical suspicion [15]. On CT, RSB often presents as either a heterogeneous mass in a spongiform pattern with an associated radio-dense linear structure and entrapped gas bubbles or, if the RSB is long-lasting, a reticular mass with a peripheral rind of calcification [9, 16]. MRI is not commonly utilized to identify RSB due to the risk of metallic fragment migration due to magnetic fields and the risk of internal tissue damage from the heat produced by radiofrequency fields [17].

Though limited in its extent, certain authors have explored the use of AI in RSB detection and recognized its potential to support human workflows (Table 1). In a study by Yamaguchi et al., the authors developed and validated a deep learning CAD system for detecting retained surgical sponges, the item found to be by far the most common RSB according to one report studying 191,168 operations at a tertiary care center [12, 18]. The software demonstrated strong performance across tests with phantom radiographs (100% sensitivity; 100% specificity), composite radiographs (97.9% sensitivity; 83.8% specificity), cadaver radiographs (97.7% sensitivity; 90.4% specificity), and normal postoperative radiographs (86.6% specificity) [18]. The software even detected sponges overlapping with bone or normal surgical matter like drains, monitor leads, and staples. Yet, these authors note that a limitation of the study was that the software only identified specific surgical sponges and could not recognize other retained surgical objects [18]. Kawakubo et al. also developed a DL model to detect retained surgical items by post-processing fused images of surgical sponges and unremarkable postoperative X-rays [19]. The authors subsequently compared the model to two experienced radiologists identifying retained surgical sponges [19]. The deep learning model had higher sensitivity and lower specificity for sponge detection compared to both human observers, suggesting its potential to support diagnostic ability by reducing the rate of missed RSBs.

**Table 1:** Characteristics of studies on AI in non-biological material imaging

Reference	Imaging Modality	Non-Biological Material Type	Model Type and Techniques	Key Quantitative Performance Measures
Yamaguchi et al. 2021	X-ray	Surgical sponges	Deep learning based on a model with a Region Proposal Network structure and end-to-end learning	100% sensitivity and 100% specificity (phantom radiographs); 97.9% sensitivity and 83.8% specificity (composite radiographs); 97.7% sensitivity and 90.4% specificity (cadaver radiographs); 86.6% specificity (normal postoperative radiographs)
Kawakubo et al. 2023	X-ray	Surgical sponges	Deep learning, neural network model	85% sensitivity and 92% specificity
Asiyanbola et al. 2012	X-ray	Surgical needles	Map-seeking circuit and modified map-seeking circuit	72.73% detection rate and 15.67% false positive rate (lower threshold for digital images analyzed by modified map-seeking circuit); 50.91% detection rate and 6.67% false positive rate (higher threshold for digital images analyzed by modified map-seeking circuit)
Sengupta et al. 2017	X-ray	Surgical needles	Rule-based, random forest, linear discriminant analysis, and neural network classifiers	75.4% sensitivity and 0.23 False Positives/image (high specificity neural network model); 86.0% sensitivity and 0.57 False Positives/image (high sensitivity neural network model)
Marentis et al. 2018	X-ray	Microtags (which can be attached to surgical instruments)	Model with candidate labeling, segmentation, feature analysis, classification, false-positive reduction, and an artificial neural network	79.5% sensitivity and 99.7% specificity (model alone); 98.5–100% sensitivity and 99.0–99.7% specificity (model combined with one of five radiologists)
Rostad et al. 2022	X-ray	Button batteries and coins	Object detector and image classifier	100% sensitivity and specificity (detection of button batteries and coins); 6/6 correctly classified button batteries, 93/95 correctly classified coins
Kara et al. 2021	X-ray	Endotracheal tubes	Convolutional neural networks: regression loss CNN function, classifier CNN, regression CNN	97.37% sensitivity and 96.89% specificity (tube detection); 0.60–0.66 cm median error for assessment of distance between carina and distal endotracheal tube tip
Brown et al. 2022	X-ray	Endotracheal tubes	Semantically embedded neural network: combines knowledge base with deep convolutional neural networks	89% overall overlay accuracy; 83% positive predictive value for alerts indicating the tube was not detected or misplaced; 98% negative predictive value for alerts indicating the tube was not detected or misplaced
Lakhani et al. 2021	X-ray	Endotracheal tubes	Deep neural network	93.9% sensitivity and 97.7% specificity for detecting when the tube-carina distance was < 1 cm
An et al. 2023	X-ray	Endotracheal tubes	Deep learning	99.2%-100% sensitivity and 94.5–98.7% specificity (tube detection in ICU radiographs); 78.9%-83.7% sensitivity and 99.1%-100% specificity (identification of improper tube position in ICU radiographs); 100% sensitivity and 99.2%-100% specificity (identification of critically improper tube position in ICU radiographs)
Wang et al. 2024	X-ray	Endotracheal tubes	Deep learning	98.8%-99.0% sensitivity and 99.2%-99.8% specificity (tube detection); 51.2%-73.3% sensitivity and 91.3%-97.6% specificity (identification of low tube position)
Lakhani et al. 2017	X-ray	Endotracheal tubes	Deep convoluted neural networks	0.99 AUC (tube detection); 0.81 AUC (identification of low tube position)
Harris et al. 2021	X-ray	Endotracheal tubes	Bounding box-based machine-learning model	99.1% sensitivity and 98.6% specificity (tube detection); 75.9% sensitivity and 88.5% specificity (identification of tube malposition); 65.9% sensitivity and 98.9% specificity (identification of severe tube malposition)
Rueckel et al. 2023	X-ray	Tracheal tubes and CVCs	Convoluted neural network	100% identification accuracy (tracheal tubes); 9.4% (manual correction of AI tracheal tube position 3-20 mm); 0.3% (manual correction of AI tracheal tube position > 20 mm); 98% identification accuracy (central venous catheters); 12.4% (manual correction of central venous catheter position 3-20 mm); 10.6% (manual correction of AI tube position > 20 mm)
Tang et al. 2023	X-ray	Endotracheal tubes, enteric tubes, and CVCs	Deep convoluted neural network	0.9999 AUC (endotracheal tube detection); 0.9983 AUC (central venous catheter detection); 0.9994 AUC (enteric tube detection); 0.9153 AUC (detection of unsatisfactory endotracheal tube position); 0.8715 AUC (detection of unsatisfactory central venous catheter position); 0.8943 AUC (detection of unsatisfactory enteric tube position)
Mallon et al. 2022	X-ray	Enteric tubes	Convoluted neural network	80% sensitivity and 92% specificity (identification of tube malposition)
Singh et al. 2019	X-ray	Enteric tubes	Deep convoluted neural network	88% sensitivity and 76% specificity (identification of tube malposition for strongest performing model)
Drozdov et al. 2023	X-ray	Enteric tubes	Artificial neural network	59% sensitivity and 87% specificity (identification of non-bronchial tube malposition); 79% sensitivity and 98% specificity (identification of bronchial tube malposition)

**Fig. 2:**
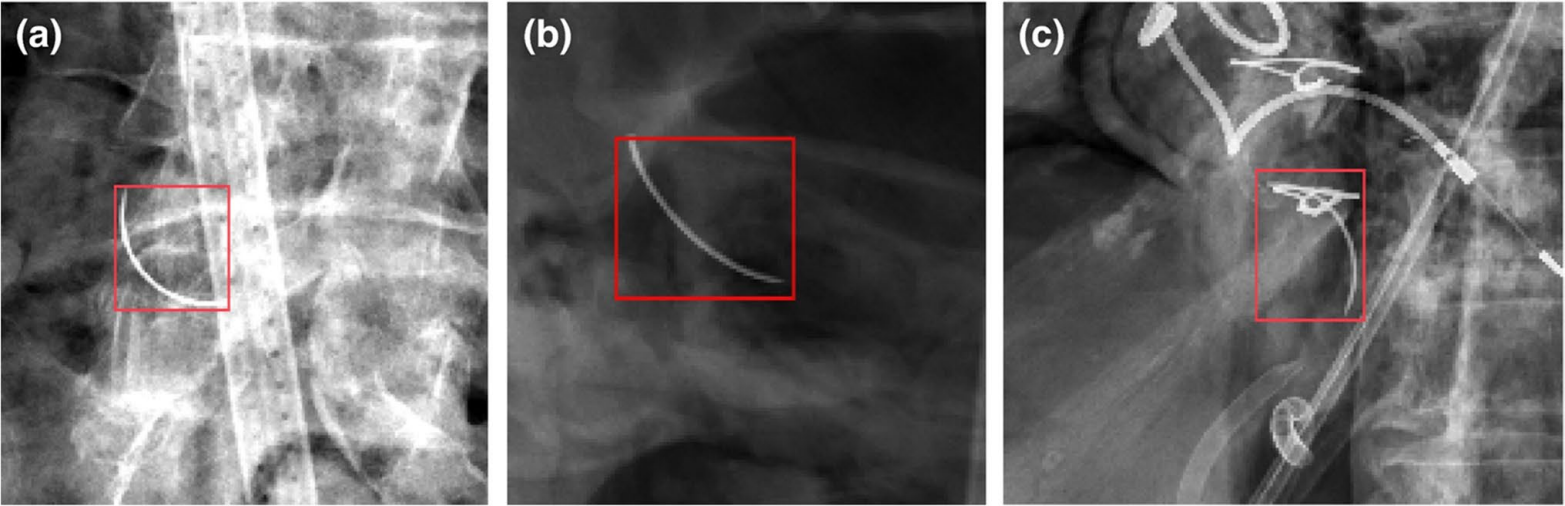
Needles of various shapes and orientations with different backgrounds that were detected by the CAD system. Figure reproduced with permission from Sengupta A, Hadjiiski L, Chan HP, Cha K, Chronis N, Marentis TC. Computer-aided detection of retained surgical needles from postoperative radiographs. Med Phys. 2017;44(1):180–191.10.1002/mp.12011

**Fig. 3:**
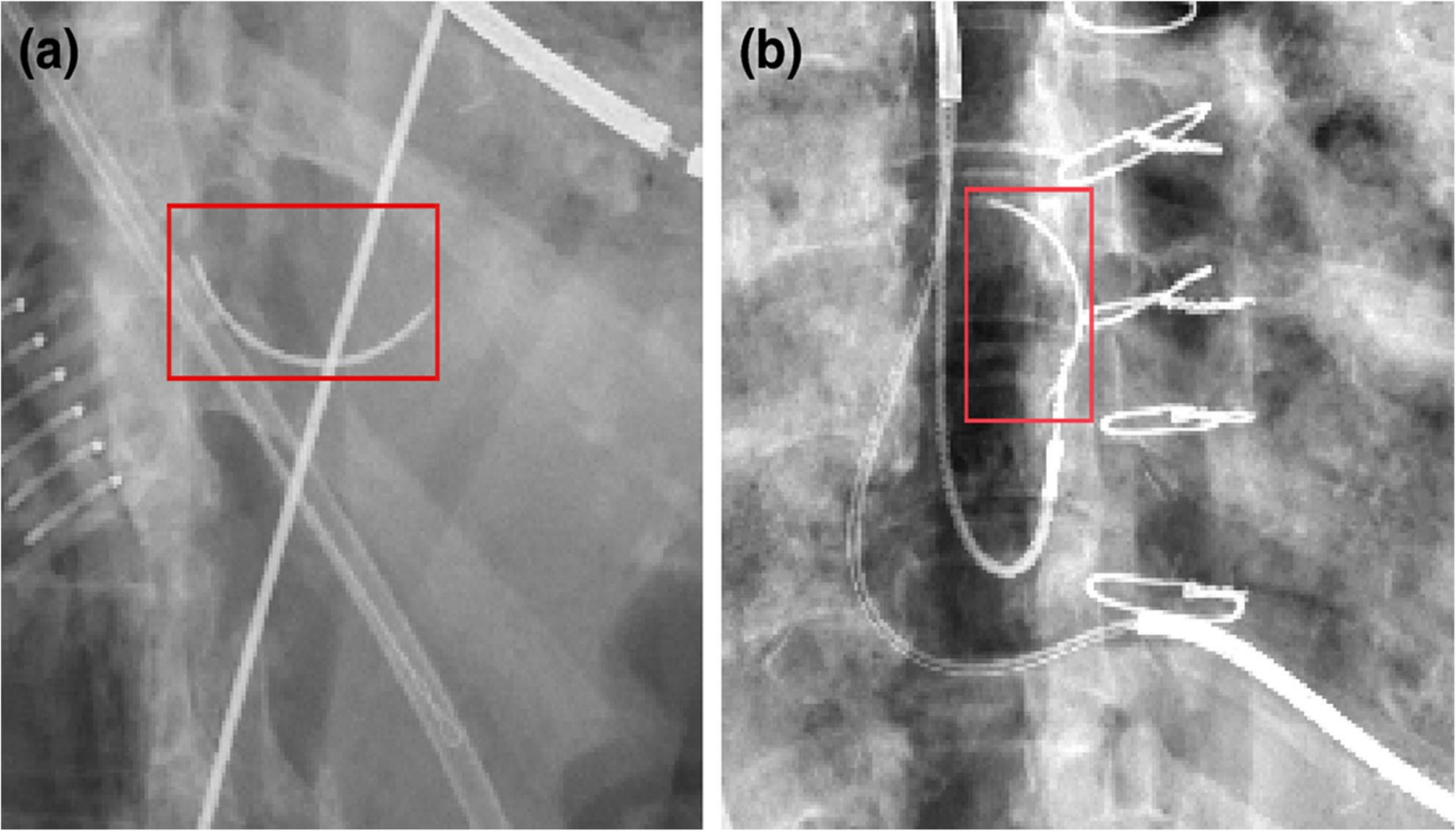
Example of needle missed by both the rule-based and the neural network-based CAD systems. Figure reproduced with permission from Sengupta A, Hadjiiski L, Chan HP, Cha K, Chronis N, Marentis TC. Computer-aided detection of retained surgical needles from postoperative radiographs. Med Phys. 2017;44(1):180–191. 10.1002/mp.12011

AI has also been employed to detect other less common RSBs, such as retained surgical needles. Accurately diagnosing retained surgical needles remains a significant issue, as certain studies report that conventional radiographs detect radiopaque needles less than 1 cm (cm) in diameter with a sensitivity of only 30% [20]. Further, surgical needles are one of the most incorrectly counted instruments [21]. In a proof-of-concept study by Asiyanbola et al., the authors generated a map-seeking circuit and a modified map-seeking circuit algorithm to detect needles in abdominal X-rays [22]. The model in this study was deployed with two detection threshold settings to analyze two sets of images and their corresponding sub-images, one set from a cassette-based X-ray machine and another from a C-arm (digital) machine [22]. The authors set these thresholds to determine when the algorithm should classify an image as containing a retained needle. The modified map-seeking circuit algorithm outperformed its unmodified counterparts with reduced computing times and higher detection rates. For the cassette-based X-ray machine, this algorithm had a detection rate of 85.19% and a false positive rate of 9.98% at the lower detection threshold and rates of 53.70% and 0.00%, respectively, at the higher threshold. For the digital machine images, the algorithm had a detection rate of 72.73% and a false positive rate of 15.67% at the lower threshold and rates of 50.91% and 6.67%, respectively, at the higher threshold [22]. Sengupta et al. also developed a series of four CAD models with rule-based, random forest, linear discriminant analysis (LDA), and neural network classifiers to detect retained surgical needles on postoperative radiographs [23]. The model was run with two modes with different decision thresholds: mode I with higher specificity and mode II with higher sensitivity. Ultimately, the authors found that the mode with high specificity yielded a neural network sensitivity and false positive (FP) rate of 75.4% and 0.23 FPs/image, respectively, and mode II with higher sensitivity had a neural network sensitivity and FP rate of 86.0% and 0.57 FPs/image, respectively [23]. Such results not only suggest AI’s ability to detect surgical needles specifically, but also can help clinicians identify what threshold can maximize algorithm sensitivity and specificity. Figure 2 demonstrates needles detected by the CAD system. In contrast, Fig. 3 showcases needles missed by this system due to overlapping structures such as bone distorting the shape of the needle.

Within RSB imaging, additional physical technological innovations can be used in conjunction with AI to enhance the effectiveness of detection furthe r[24]. In a study by Marentis et al., the authors demonstrated the efficacy of CAD in detecting radiopaque micro-tags, which can be attached to sponges and other surgical instruments [25]. In the detection of these micro-tags, the high-specificity CAD system had a sensitivity of 79.5% and a specificity of 99.7%, and after the use of this CAD system in conjunction with one of five radiologists, sensitivity ranged from 98.5–100% and specificity from 99.0–99.7% [25]. This data ultimately shows the high utility of combining a CAD system with a radiologist to complement one another in detecting RSB.

## Penetrating and open injuries

Another application of AI that will be discussed involves imaging of penetrating wounds. This class of injuries is caused by objects that pierce and penetrate the skin to create an open wound [26]. Firearms and sharp objects are among the most common causes of these injuries [26]. One report estimated that in the United States alone, from 2009–2017, an annual average of more than 85,000 emergency department visits annually were attributable to nonfatal firearm injury in addition to 34,538 deaths [27]. Additionally, the CDC estimates that annually in the United States, cut or pierce wounds are responsible for over 1.8 million nonfatal injuries, along with over 3,000 deaths [28]. Prompt diagnosis of penetrating wounds is crucial to facilitate effective management and intervention.

While large, superficial foreign objects are often detected by palpation, imaging plays a role when detecting smaller foreign bodies in patients with open wounds or penetrating injuries. Ultrasound, for instance, can be highly useful in identifying a radiolucent foreign body and assisting with object removal [29]. On ultrasound, foreign bodies disrupt the homogenous echogenicity inherent in soft tissue and thus often present hyperechoic compared to surrounding tissue [30]. Over time, hypoechoic rings can form around the foreign object, which indicates the development of inflammatory processes. Some advantages of ultrasonography include its ability to image dynamically and provide timely access compared to other modalities [31]. In superficial tissues, US may even offer higher resolution than X-ray or CT. However, its effectiveness can be minimized when imaging deeper tissue, as ultrasound’s acoustic waves only penetrate to a certain depth. This may be further limited by bone or air obfuscation of the region of interest [32]. Yet, US is restricted by its dependence on operator skill and its limitations in detecting foreign bodies of smaller sizes [33]. Certain authors reported a decline in foreign body identification by almost 20% when the size of the foreign object decreased from 2 to 1 mm [31]

**Fig. 4:**
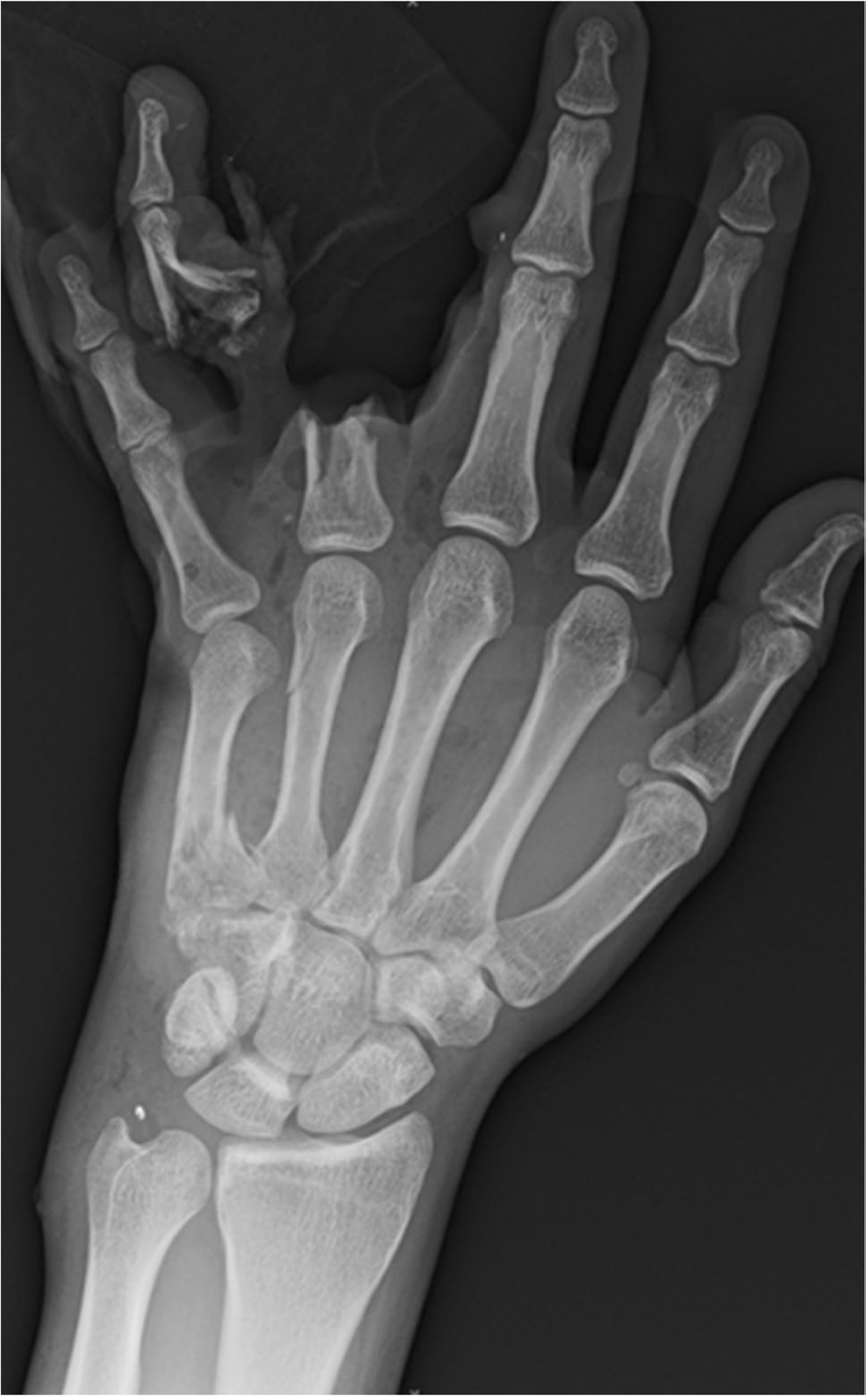
23-year-old male who presented with a gunshot wound to the left hand. Other than the amputation of the fourth finger and multiple fractures, the X-ray demonstrates small retained metallic bullet fragments about the 3rd proximal interphalangeal joint and ulnar styloid process

Conventional radiography can also detect foreign bodies from penetrating injuries, but the advent of more advanced imaging modalities makes it less commonly utilized [34]. This technique remains limited because its detectability depends on the density contrast with surrounding tissue, making it difficult to detect objects such as wood or plastic due to density similarities with soft tissue and graphite and gravel due to density similarities with bone [32, 35]. However, radiographs can still be used to identify retained metal, such as metallic bullet fragments (Fig. 4).

Conversely, CT is the first-line modality in imaging penetrating injuries due to its high specificity and sensitivity and its ability to acquire multiplanar images relatively quickly [36, 37]. CT angiography (CTA) is also often utilized to detect suspected vascular injury. Ultimately, the multiplanar nature and re-constructability of CT and CTA aid in the detection of injuries within the tissue, as well as help predict probabilistic injuries [38]. While CT and CTA excel in imaging penetrating injuries, AI introduces a promising avenue for further enhancing accuracy and efficiency in detecting such injuries.

Presently, there is limited literature regarding the use of AI in imaging penetrating wounds. A series of models, TraumaSCAN and TraumaSCAN-Web, have employed three-dimensional (3D) anatomical models in conjunction with patient signs, symptoms, and imaging findings to estimate the likelihood of injury to anatomic structures as well as the probability of subsequent conditions using Bayesian networks [39–41]. However, these models do not use AI to evaluate the images themselves; instead, they rely on human assessment to output a variable, which serves as an input for the model [42]. Thus, it is evident that further development of AI models is necessary before CAD systems are implemented within clinical practice. Yet, integrating AI with other clinical variables presents the potential for rapid, streamlined clinical evaluation in urgent, high-acuity cases of penetrating wounds.

Before AI can be confidently utilized for the imaging of penetrating wounds, a number of challenges must be addressed. First, different models must be developed for each existing imaging modality. Second, the diversity of objects causing penetrating injuries, coupled with the multitude of potential locations on the body that an object can penetrate, require large, standardized datasets to train a potential model [43]. Furthermore, some penetrating objects may splinter within the body or may induce bone fragments, which can have varied trajectories as secondary projectiles [43]. Other challenges involve cases where penetrating objects have left the body. Thus, it is difficult for AI models to ascertain the penetrating object’s tract within the body and the subsequently injured tissues [43]. However, even if not directly involved in the identification of the object’s track or injured tissues, AI models still have the potential to augment such clinical workflows through image enhancement or reconstruction.

## Catheter/tube malposition

Endotracheal tubes (ETT), enteric tubes, and central venous catheters (CVCs) are devices commonly employed in emergency or intensive care settings to provide and deliver care. However, malpositioning of these devices can result in adverse outcomes, either through direct harm from improper insertion or the inability to provide treatment. The malposition of endotracheal tubes, enteric tubes, and central venous catheters is estimated to occur in 5–28%, 3–20%, and 2–7% of cases, respectively [44]. Ultimately, an automated method to interpret catheter and tube malposition may allow for earlier identification and reduce the detrimental effects of an improperly placed tube.

Chest radiography is the preferred imaging technique to confirm the proper positioning of these devices after placement, mainly due to its low cost and wide availability [45]. Portable X-rays are often employed in emergency departments or intensive care units (ICU), although these often result in images with low contrast and high noise [45]. Radiographs should also be obtained after any positioning changes in support devices, after bedside procedures, and if a patient experiences an acute change in clinical status [46]. In addition to X-ray, ultrasound has emerged as another rapid and viable alternative with high diagnostic accuracy [47]. However, ultrasound has limitations in cases with unusual airway anatomy, cervical collars, neck edema, subcutaneous emphysema, or neck masses [48].

Currently, substantial research is occurring regarding the use of AI in tube/catheter malposition, particularly endotracheal tube detection and position localization [49]. While the data regarding endotracheal tube detection and critical tube malpositions (ETT-carina distance < 1 cm) is strong across studies, the models identifying subtle malpositions are weaker. In a model developed by An et al., the sensitivity and specificity for detection of critical tube position (ETT-carina distance < 1 cm) amongst ICU images was 100% and 99.2%-100%, respectively, whereas detection of less critical malpositions resulted in sensitivities and specificities of just 72.5%-83.7% and 92.0%-100% [50]. Lakani et al. reported similar findings with a sensitivity of 93.9% and specificity of 97.7% for differentiating ETT-carina distance of less than 1 cm, but the sensitivity and specificity were 66.5% and 99.2%, respectively, for differentiating ETT-carina distance > 7 cm [51]. Such results indicate that a complementary rather than entirely independent role may be most appropriate for such models [52]. If AI models can alert ICU physicians and radiologists when the endotracheal tube is improperly positioned, clinicians can quickly evaluate the need for ETT repositioning and assess patient safety.

AI has also been used to detect central venous catheter malposition. A model developed by Rueckel et al. reported that chest radiographs with improperly positioned CVCs were identified with an area under the receiver operating characteristic curve (AUC) of > 0.93–0.96 [53]. Tang et al.’s model achieved an AUC of 0.8715 for detecting unsatisfactory tube position [54]. However, the application of AI position detection with this class of devices presents additional challenges compared to endotracheal tubes. For example, it is more difficult to define optimal CVC position, and CVC insertions may occur through different veins. Additionally, there are a variety of mimicking objects, such as pacemaker wires, electrocardiogram (ECG) electrodes, and sheaths [53]. In their analysis of various central venous catheter subgroups, Tang et al. also noted that their model found it more challenging to detect peripherally inserted CVCs when compared to other subtypes, including dialysis catheters and jugular and subclavian lines [54]. This is likely a consequence of the thinner lines of peripherally inserted central catheters, as well as the more variable, peripherally located tips compared to other subtypes [54]. These results highlight the need for specific models to be developed for certain subtypes of catheters or tubes.

**Fig. 5:**
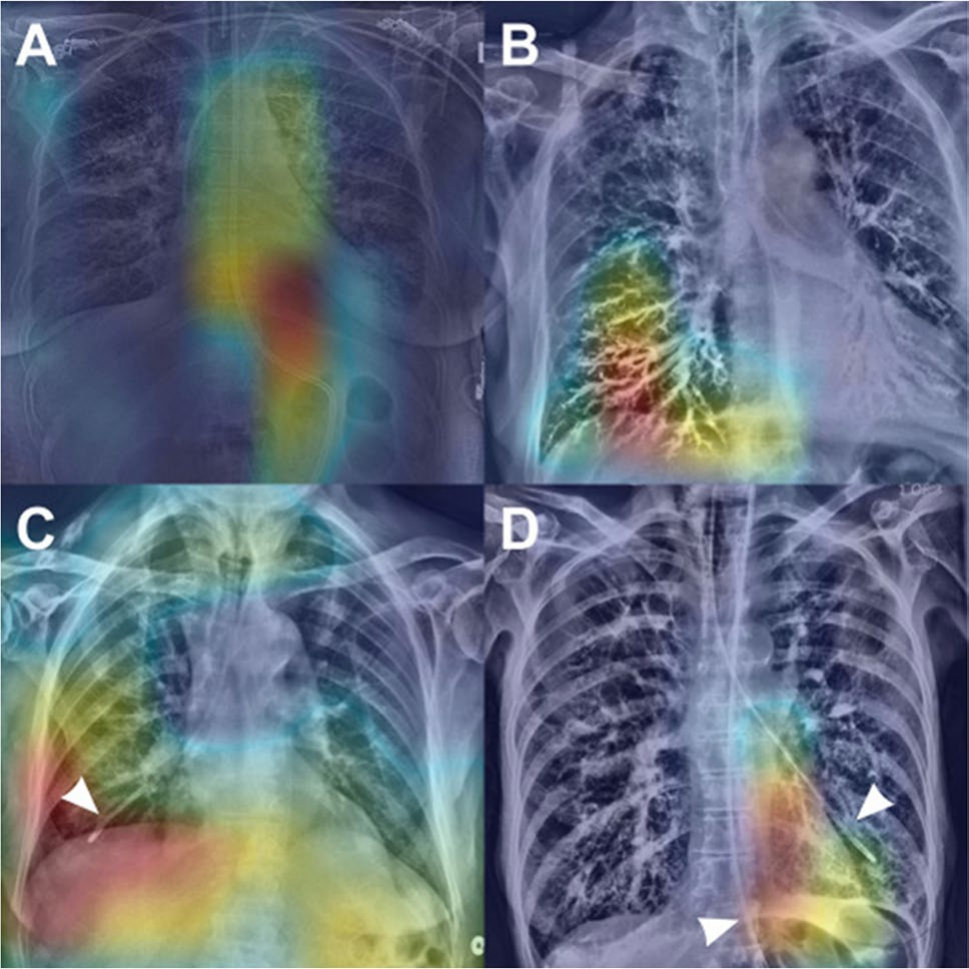
Failure analysis using class activation maps that highlight regions of interest within each radiograph. A. Correct classification of a safe enteric tube position shows maximum activation values along the course of the esophagus and stomach. B. Incorrectly classification of a safe enteric tube position (false positive), with high activation in the right lower zone caused by linear opacification due to aspiration of barium. C. Correct classification of an enteric tube that is misplaced within the right lower lobe airways. D. Incorrect classification of a misplaced enteric tube within the left lower lobe (false negative). Misclassification may be due to the presence of a safely positioned enteric tube that enters the stomach. Figure reproduced with permission from Mallon DH, McNamara CD, Rahmani GS, O'Regan DP, Amiras DG. Automated detection of enteric tubes misplaced in the respiratory tract on chest radiographs using deep learning with two-centre validation. Clin Radiol. 2022;77(10):e758-e64

Research has also explored the use of AI in detecting enteric tubes, though the performance of these models leaves room for improvement (Table 1). Mallon et al.’s algorithm detected critically misplaced enteric tubes with sensitivities and specificities of 80% and 92%, respectively [55]. Other authors reported sensitivities of 100% and specificities of 76%, respectively, in identifying enteric tube malposition [56]. When used in conjunction with human readers, one model tested by Drozdov et al. increased the confidence of junior emergency medicine physicians and their interpretative capabilities [57]. When junior physicians were given a second opinion from this AI model regarding enteric tube placement, sensitivity and specificity increased from 96 to 100% and from 69 to 78%, respectively [57]. However, it is essential to address the elevated rate of false positives and negatives reported by these algorithms. Analysis of one model noted false positives due to ECG leads and endobronchial barium and false negatives when multiple tubes were present [55]. Figure 5 showcases class activation maps utilized to conduct failure analysis for the false positives and negatives reported. Additionally, some models highlighted many irrelevant features, a frequent flaw of algorithms that analyze the whole image. Further, applying segmentation techniques to circumvent this issue adds complexity and room for other sources of error [55, 56]. At present, the high number of false positives and negatives associated with these models minimizes their efficacy but highlights their potential role as a complementary tool to human readers.

## Foreign Body Ingestion/Aspiration

Foreign body ingestion represents a significant clinical problem that can manifest itself in a variety of forms. Ingestion of foreign bodies is particularly prominent among those with psychiatric or neurological disorders as well as young children, and it is estimated that between 1995 and 2015, 795,074 patients under the age of six years old presented to the ED for foreign body ingestion [58–60]. Among the most commonly ingested items are coins, toys, jewelry, batteries, and bones, including fish bones [58]. One of the major consequences of foreign body ingestion is aspiration, a complication often seen among young children [59]. Globally, it is estimated that from 1990–2019, foreign body aspiration had an incidence of 109.6 per 100,000 children under five years old[61]. Like foreign body ingestion, the most commonly aspirated objects include batteries, coins, and other inorganic objects, though organic objects and food items are far more frequent causes [62].

**Fig. 6:**
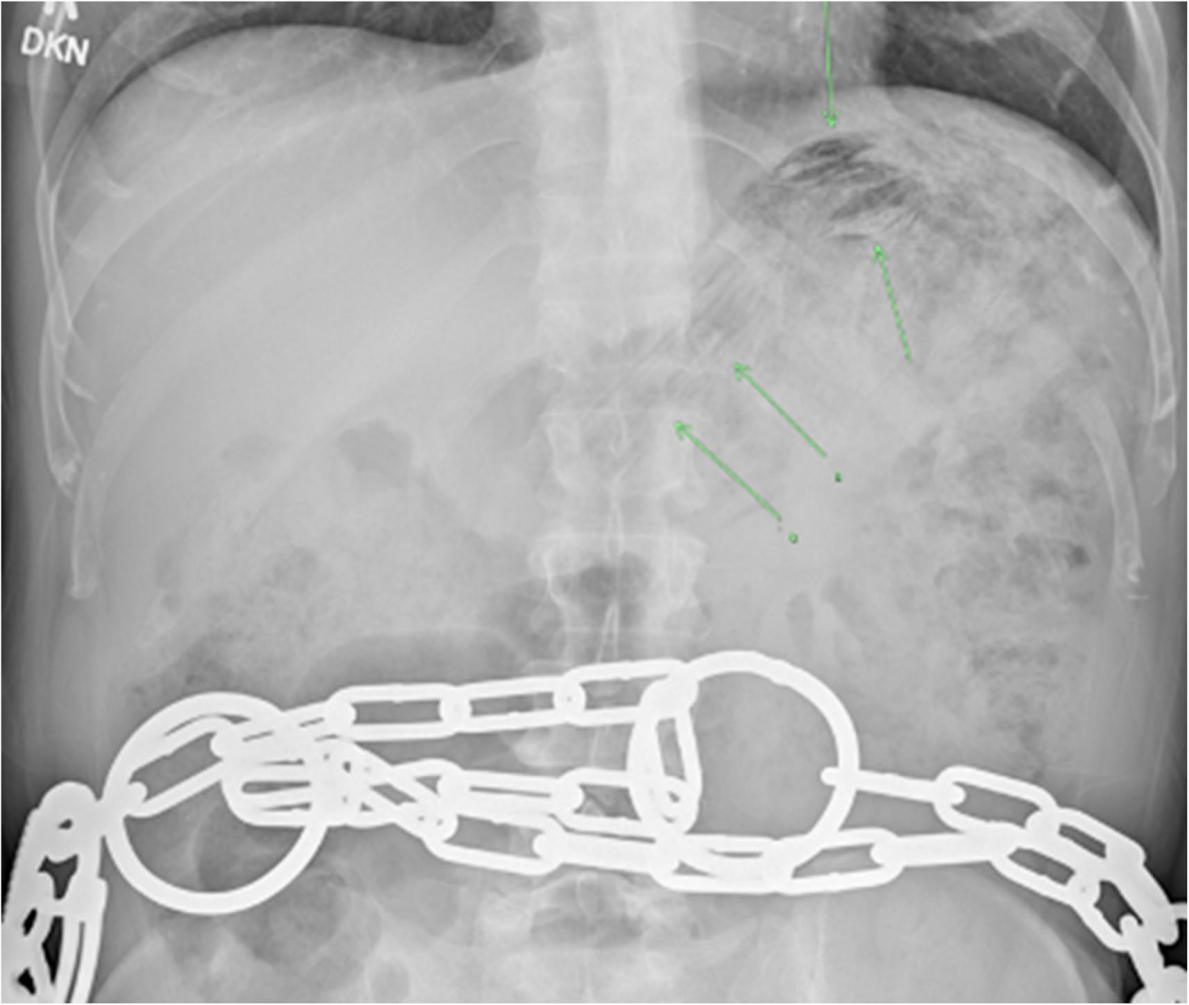
51-year-old male with past medical history of schizoaffective and schizotypal personality disorder and multiple prior foreign body ingestions. Abdominal radiograph shows multiple regular radiolucencies projecting over the gastric fundus and body in the left upper abdominal quadrant (green arrows), concerning for a radiolucent foreign body. This was initially missed due to the small difference in density between soft tissue and plastic bags. Upper GI endoscopy found multiple plastic bags, which were successfully removed

In order to detect foreign body ingestion, various imaging modalities can be utilized. Ultrasound is beneficial in the detection of radiolucent foreign bodies and for imaging in the pediatric population [63]. Radiographs are commonly used for initial diagnosis due to their widespread availability and ability to detect foreign bodies cheaply and rapidly. Further, radiographs can help quickly rule out aspirated foreign objects [64]. This technique is often the first-line imaging modality to detect radiopaque objects, yet it is imperative to note that a negative X-ray can only rule out retained radiopaque materials but not retained radiolucent foreign bodies [65]. Figure 6 represents the X-ray findings of a patient who ingested multiple radiolucent plastic bags, which were initially overlooked due to a small difference in density between the plastic bags and soft tissue. Some common radiolucent foreign bodies include chicken and fish bones, plastic, wood, and small metal objects [66]. There is also often a role for serial X-ray imaging if the object is most likely to pass without intervention.

Compared to X-ray, CT has a higher sensitivity in imaging foreign objects. This technique allows for the detection of radiopaque objects such as metal, stone, and glass and can also detect objects, including plastics, wood, or other organic materials [32]. The 3D rendering of cross-sectional CT images also allows for enhanced localization and detection, which may aid in removing the foreign body [67]. Further, 3D models help prevent the obscuring of foreign objects by bone[33]. However, CT is often not the modality used for initial imaging due to the high level of radiation, its cost, and low sensitivity for the detection of radiolucent materials [33].

Lastly, MRI is typically the most expensive, timely, and least widely available of the major imaging modalities, leading to its limited use in foreign body detection [33]. Additionally, it can be challenging to ascertain an object’s ferromagnetic properties. Thus, significant safety concerns exist with the potential interaction between the magnetic field and ferromagnetic foreign bodies. However, MRI is vital in the imaging of radiolucent objects, as it can visualize tissues not apparent on ultrasound [32].

**Fig. 7:**
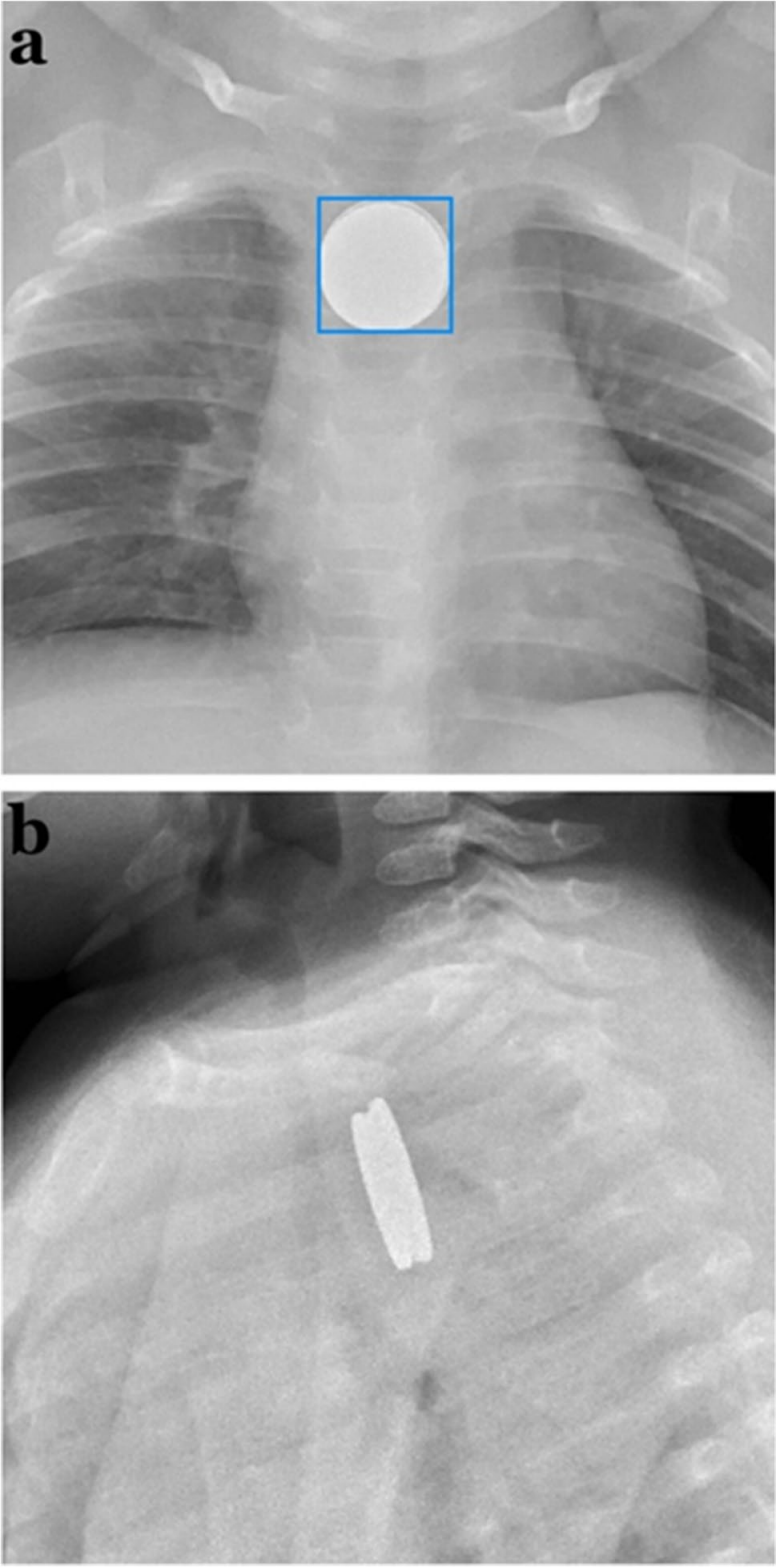
An 11-month-old girl with a stacked button battery and coin in her proximal esophagus. A: An anteroposterior chest radiograph shows the stacked button battery and coin were detected but classified as a coin. B: The lateral radiograph view shows the stacked button battery and coin. Figure reproduced with permission from Rostad, B. S., E. J. Richer, E. L. Riedesel and A. L. Alazraki (2022). "Esophageal discoid foreign body detection and classification using artificial intelligence." Pediatr Radiol 52(3): 477–482

Despite the common occurrence of foreign body ingestion and aspiration, there is a dearth of literature regarding the use of AI for imaging in this capacity. The few articles published on AI’s role in foreign body ingestion and aspiration emphasize the advantage of CAD systems in not only detecting foreign objects but also classifying them. In a study by Rostad et al., the authors developed two AI models for analysis of pediatric esophageal radiographs, one which aimed to detect discoid foreign bodies and a subsequent one which aimed to classify objects such as coins or button batteries [68]. As button batteries in the esophagus require emergent endoscopic removal, the presence of coinlike objects on radiographs must be differentiated [69]. Ultimately, the authors reported that the object detector identified all foreign bodies with 100% specificity and 100% sensitivity. The image classifier also demonstrated strong performance, classifying 6/6 (100%) button batteries as such, 93/95 (97.9%) of the coins as such, and 2/95 (2.1%) of the coins as button batteries [69]. Outside of these instances, there were only two cases incorrectly classified as coins: a stacked button battery and coin (Fig. 7), as well as two stacked coins.

## Limitations of AI imaging

Yet, these cases of incorrect object classification illustrate an essential limitation when applying AI to foreign body imaging. First, the model’s ability to detect objects relies on the images encountered during the training data set. Thus, the model will not be able to identify and classify foreign objects it has not previously encountered. This was particularly evident in the model developed by Kawakubo et al., as the software only identified specific surgical sponges for which the model was trained and could not recognize other retained surgical objects [19]. Moreover, the breadth and variety of training datasets, encompassing objects in diverse orientations and forms, are crucial for AI's ability to detect foreign bodies. Further, gaining access to expansive datasets remains challenging given patient data and privacy concerns, though systems are being developed to circumvent this [70]. Complicating the matter is the fact that models must be developed and trained for each imaging modality, a particularly significant issue when the object composition is unknown and the most effective imaging technique is not immediately apparent. Lastly, there are challenges associated with training software to recognize and/or classify heterogeneous objects. Objects of uniform size and shape, such as surgical equipment or medical tubes and lines, are far easier to train models to recognize compared to commonly aspirated or ingested objects like fish bones, toys, or jewelry of varying size and composition. Thus, it is unsurprising that one of the first reports demonstrating AI’s utility in imaging ingested foreign bodies has been with coins and button batteries: objects of uniform shape and size.

## Conclusion

Despite AI’s enormous potential in foreign body detection, current applications have thus far been in research settings, often training and validating models on devised images such as those with cadavers or fusion images. Before the widespread deployment of AI systems, these models must be trialed on natural datasets to ensure real-world clinical utility and performance. Though significant legal hurdles surrounding liability and tort law remain that may limit AI’s potential use, the ongoing advancements in the field augment its clinical utility and potential [71]. Despite these challenges, the advancements in AI technology, coupled with collective efforts to obtain diverse and comprehensive datasets, offer a promising trajectory for the future of medical imaging in foreign body analysis. Further, the integration of AI in clinical practice has the potential to alleviate radiologist workload, enhance their efficiency, and reduce diagnostic errors. As the field of medical imaging continues to progress, the collaboration between AI and radiology may ultimately enhance diagnostic precision and patient care.
